# 2383: Electrical stimulation to the subcallosal cingulate and amygdala drive shifts in affective bias across patient populations

**DOI:** 10.1017/cts.2017.228

**Published:** 2018-05-10

**Authors:** Kelly Rowe Bijanki, Jon Willie, Helen Mayberg, Jess Fiedorowicz, Christopher Kovach, Cory Inman, Andrea Crowell, Robert Gross, Daniel L. Drane

**Affiliations:** Department of Neurology, Emory University School of Medicine, Atlanta, GA, USA

## Abstract

OBJECTIVES/SPECIFIC AIMS: Deep brain stimulation is currently being evaluated as an experimental therapy for various psychiatric disorders, as well as being investigated as a method for mapping emotional brain functions. This growing area of research requires sensitive measures to quantify effects of stimulation on emotional processing. The current study examined the effects of acute stimulation to 2 limbic regions—the subcallosal cingulate (SCC) and the amygdala—on bias in the perception and evaluation of emotional facial expressions. We hypothesized that transient electrical stimulation to the limbic system would produce acute reductions in negative bias, consistent with its antidepressant effects in patients with severe depression. METHODS/STUDY POPULATION: The current study uses a novel affective bias task, developed to rapidly and covertly quantify emotional state. Over 4–6 minutes, patients rate the intensity and valence of static images of emotional facial expressions. We examined effects of electrical brain stimulation in 2 groups: patients with treatment-refractory depression undergoing SCC DBS therapy, and epilepsy patients undergoing amygdala stimulation via stereo-EEG electrodes during inpatient intracranial monitoring. DBS patients completed the task under stimulation and sham conditions during monthly visits over the first 6 months of therapy, as well as daily during a 1 week, blinded period of DBS discontinuation at the 6-month time point. Epilepsy patients completed the task under stimulation and sham conditions at a single visit. Mixed linear models and paired-samples *t*-test were used to investigate effects of stimulation as well as depression scale scores on affective bias ratings. RESULTS/ANTICIPATED RESULTS: Four SCC DBS patients showed significant effects of stimulation (*p*<0.0001) and depressive state (*p*<0.0001) on affective bias scores across 6 months of chronic DBS therapy, where emotional faces were perceived as less sad with stimulation ON, as well as during visits in which patients were nondepressed (typically later in the treatment course). Furthermore, 2 DBS patients showed rapid negative shifts in bias following acute blinded discontinuation of chronic stimulation, an effect which persisted over the 1-week period of discontinuation (*t*_29_=−2.58, *p*=0.015), in the absence of any self-reported change in mood. Likewise, 6 epilepsy patients showed significant positive shifts in affective bias with acute amygdala stimulation (*t*_5_=−4.75, *p*=0.005). Current analyses are investigating electrophysiological, autonomic and facial motor correlates to affective bias in these patients. DISCUSSION/SIGNIFICANCE OF IMPACT: Affective bias has revealed rapid, significant changes with stimulation at 2 limbic targets—one a white matter hub and one a nuclear subcortical structure—suggesting the task’s utility as an emotional outcome measure in brain stimulation studies. These stimulation-sensitive measures may provide a new metric to track treatment response to deep brain stimulation therapy for affective disorders. Future studies will determine whether affective bias can predict neuropsychiatric complications in patients undergoing stimulation mapping of brain circuitry ahead of resection surgery for epilepsy.

